# Juvenile hormone regulation of female reproduction in the common bed bug, *Cimex lectularius*

**DOI:** 10.1038/srep35546

**Published:** 2016-10-20

**Authors:** Hemant Gujar, Subba Reddy Palli

**Affiliations:** 1 Department of Entomology University of Kentucky, Lexington, KY 40546-0091, USA

## Abstract

To begin studies on reproduction in common bed bug, *Cimex lectularius*, we identified three genes coding for vitellogenin (Vg, a protein required for the reproductive success of insects) and studied their hormonal regulation. RNA interference studied showed that expression of Vg3 gene in the adult females is a prerequisite for successful completion of embryogenesis in the eggs laid by them. Juvenile hormone (JH) receptor, Methoprene-tolerant (Met), steroid receptor coactivator (SRC) and GATAa but not ecdysone receptor (EcR) or its partner, ultraspiracle (USP) are required for expression of Vg genes. Feeding and mating working through Vg, Met, SRC, EcR, and GATAa regulate oocyte development. Knockdown of the expression of Met, SRC, EcR, USP, BR-C (Broad-Complex), TOR (target of rapamycin), and GATAa in female adults resulted in a reduction in the number eggs laid by them. Interestingly, Kruppel homolog 1 (Kr-h1) knockdown in the adult females did not reduce their fecundity but affected the development of embryos in the eggs laid by females injected with Kr-h1 double-stranded RNA. These data suggest that JH functioning through Met and SRC regulate both vitellogenesis and oogenesis in *C. lectularius*. However, JH does not work through Kr-h1 but may work through transcription factors not yet identified.

Ecdysteroids (20-hydroxyecdysone, 20E, the most active form) and juvenile hormones (JH) regulate many aspects of insect life including development and reproduction. They act as gonadotrophic hormones in the adult female insects and regulate vitellogenesis and oogenesis^1^,^2^,^3^,^4^. The hormonal control of female reproduction varies widely among insect species; in some insects, only one of these two hormones regulate expression of vitellogenin (Vg) genes. While in the other insects including mosquitoes and fruit flies both these hormone are known to regulate this process^3^,^5^,^6^,^7^. Both JH and ecdysteroids have been shown to regulate female reproduction in hymenopteran and lepidopteran insects^8^,^9^,^10^,^11^. In the mosquito *Aedes aegypti*, a blood meal induces synthesis of ecdysteroids in the ovary; amino acids and ecdysteroids along with ecdysteroid-induced transcription factor (hormone receptor 3), JH and its receptor regulate four waves of gene expression/repression between 0–72 hours after blood meal^12^. Thus, nutrition, as well as JH and ecdysteroids, control reproduction in this mosquito species. In *Tribolium castaneum*, 20E plays a major role in oocyte maturation^13^, application of JH but not an injection of 20E induced Vg gene expression^14^. Microarray and RNAi studies revealed that JH regulates Vg gene expression in the fat body working through its receptor Methoprene-tolerant protein (Met) and coactivator, steroid receptor co-activator (SRC)^15^.

In locusts and cockroaches, JH has been shown to play a major role in the induction of Vg genes^2^,^16^. Application of JH induces Vg gene expression in the fat body dissected from locust and cockroach^17^,^18^,^19^,^20^,^21^. Recent studies in the cockroach, *Diploptera punctata* showed that knockdown of Met resulted in an arrest of oocyte development and Vg gene expression^22^. In the linden bug, *Pyrrhocoris apterus*, knockdown of Met or Taiman, but not Kr-h1, blocked ovarian development and suppressed vitellogenin gene expression in the fat body suggesting that JH may rely on a common receptor but different partners in transduction of JH signals in the regulation of development and reproduction^23^. The increase in amino acid concentration after feeding induces Vg gene in the fat body in concert with JH biosynthesis rather than JH action in *Nilaparvata lugens*^24^.

Nutrition also plays a major role in the regulation of reproduction. Nutrition signals functioning through insulin and TOR pathways are shown to regulate reproduction in many insects including *Ae. aegypti*^25^,^26^,^27^, *T. castaneum*^28^,^29^ and *Blattella germanica*^30^. In this paper, we report on the identification of three vitellogenin genes, studies on their function in reproduction as well as regulation of their expression.

## Results

### Vitellogenin genes and proteins

**Figure 1:**
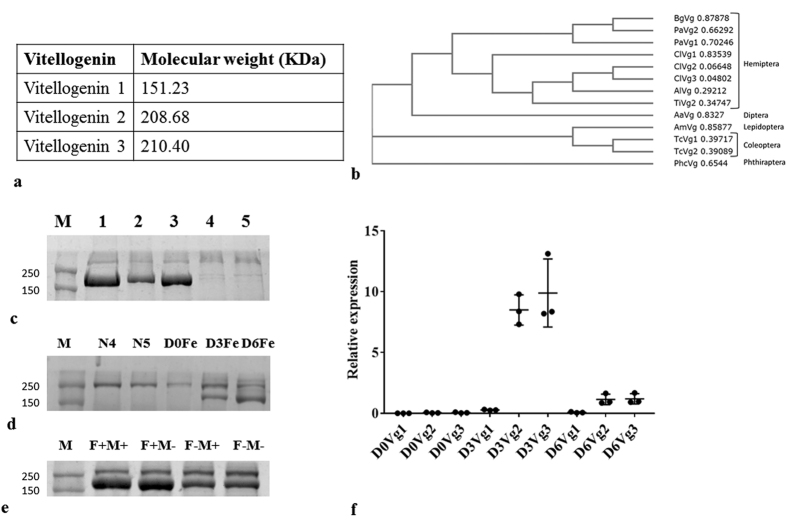
Identification of three vitellogenin genes and proteins. (**a**) Three vitellogenin genes (Vg1, Vg2, and Vg3) were identified in *Cimex lectularius* genome. The predicted protein molecular weight for the Vgs are shown. (**b**) The phylogenetic tree drawn based on the predicted Vg protein sequences from different insects is shown. The tree was created using clustal W. Bg, *Blattella germanica*; Pa, *Periplaneta americana*; Cl, *Cimex lectularius*; Al, *Apolygus lucorum;* Ti, *Triatoma infestans;* Aa, Aedes aegypti; Am, *Apis mellifera*; Tc, *Tribolium castaneum*; Phc, *Pediculus humanus corporis.* (**c**) Hemolymph proteins collected from insects injected with VgdsRNA were resolved on SDS-PAGE. M, molecular weight markers; 1, dsGFP; 2, dsVg1 and 2; 3, dsVg1 and 3; 4, dsVg2 and 3; 5, dsVg1, 2 and 3. (**d**) Vg protein profiles in the hemolymph collected from day 2 4^th^ instar (N4), day 3 5^th^ instar (N5), day 0 adult female (D0Fe), adult female three days after emergence (D3Fe) and adult female 6 days after emergence (D6Fe). (**e**) Hemolymph proteins collected from adult females on the 3^rd^ day after feeding and mating (F+M+), feeding only (F+M−), mating only (F−M+) and no feeding no mating (F−M−) were resolved on SDS-PAGE. (**f**) Vg mRNA levels increase on the 3^rd^ day after adult emergence. Total RNA isolated from whole body of adult females on 0, 3 and 6 days after adult emergence was converted to cDNA. These cDNAs and primers are targeting Vg1, Vg2 and Vg3 were used to quantify relative mRNA levels using ribosomal protein 8 (rpl8) mRNA levels for normalization.

Three vitellogenin mRNAs have been identified in the genome and transcriptome of *C. lectularius*. The ~200 KDa protein band was identified as Vg2 and Vg3 using MALDI-TOF/TOF (Fig. S1). Vg1 is 1321 aa long, contains the DUF1943 and the VWD domain and is similar to that found in other hemipteran insects (Fig. 1a,b). Vg2 (1854 aa long) and Vg3 (1868 aa long) also contain Vitellogenin_N, DUF1943, and the VWD domain and are closer to Vg2 of *Triatoma infestans* (Fig. 1a,b). The Vg in *C. lectularius* is closely related to those in the other hemipterans as shown by the phylogenetic tree (Fig. 1b).

To determine the relative contribution of three Vg genes to Vg protein levels in the hemolymph, RNAi was used to knockdown of either one or more of Vg genes. 10 μg of total hemolymph protein collected from insects injected with dsGFP (Fig. 1c. lane 1, all three Vg proteins), dsVg1 and dsVg2 (Fig. 1b. lane 2, Vg3 protein), dsVg1 and dsVg3 (Fig. 1b, lane 3, Vg2 protein), dsVg2 and dsVg3 (Fig. 1c, lane 4, Vg1 protein) and dsVg1, dsVg 2 and dsVg3 (Fig. 1c, lane 5, no Vg protein) were resolved on SDS-PAGE. As shown in Fig. 1c, no Vg protein band was detected after knockdown of Vg2 and Vg3 or Vg1, Vg2 and Vg3 suggesting that Vg2 and Vg3 constitute the majority of the Vg protein detected in the hemolymph.

Hemolymph collected from 4^th^ instar (N4), 5^th^ instar (N5) nymphs, and adult females soon after emergence (D0), three (D3) and six days (D6) after emergence resolved on SDS-PAGE. No Vg was detected in N4, N5, and newly emerged adults but Vg protein was detected in D3 and D6 adults (Fig. 1d). To study the effect of nutrition and mating on Vg synthesis, total protein from the hemolymph was collected from adults on the third day after blood feeding and mating (F+M+), only feeding (F+M−), only mating (F−M+) and no feeding and no mating (F−M−). Although all the groups showed the presence of Vg protein, Vg was more abundant in the first two groups (F+M+ and F+M−) as compared to the last two groups (F−M+ and F−M−) (Fig. 1e). The maximum levels of Vg mRNA were detected in the females at three days after adult emergence (Fig. 1f).

### The function of Vg

**Figure 2:**
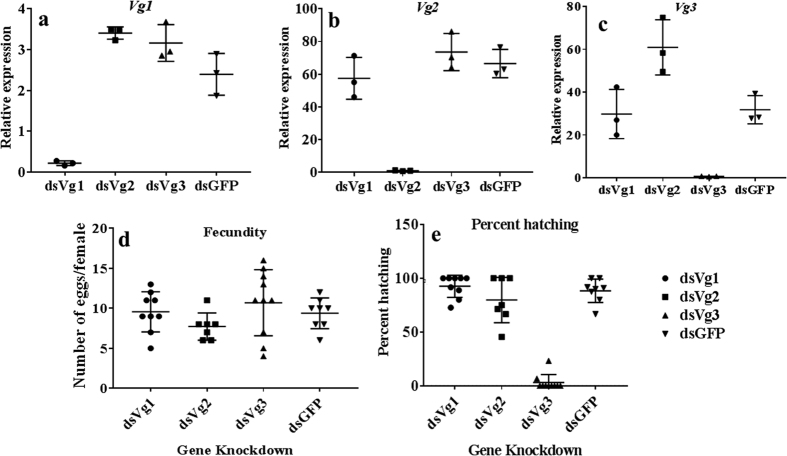
The function of three vitellogenins. To determine the function of three Vgs in *Cimex lectularius,* adult females were injected with dsGFP, dsVg1, dsVg2 or dsVg3. Total RNA was isolated and used in qRT-PCR to determine relative mRNA levels of Vg1 (**a**), Vg2 (**b**) and Vg3 (**c**). (**d**) Effect of vitellogenin knockdown on fecundity. Fecundity in females injected with dsGFP, dsVg1, dsVg2 or dsVg3 and mated with uninjected males was recorded. Insects were fed only once for 15 min. they were then allowed to mate for two days after which the males were removed. The number of eggs were counted 10 days after mating. (**e**) Effect of vitellogenin knockdown on percent hatching. Percent hatching was determined seven days after eggs were laid by female injected with dsGFP, dsVg1, dsVg2 or dsVg3 and mated with untreated males.

To determine the function of Vg proteins in reproduction, fecundity was determined in adult females injected with Vg dsRNA. As shown in Fig. 2a–c, the Vg dsRNAs designed to target each of the three Vg genes specifically knockdown the target gene (70 = 90% knockdown of target gene and no significant effect on the expression of the other two Vg genes). There was no difference in the number of eggs laid by dsVg injected, and control insects injected dsGFP (Fig. 2d). However, the significantly lower number of eggs laid by females injected with dsVg2 and dsVg3 hatched when compared to the hatching observed in Vg1 knockdown and control insects (Fig. 2e). These data suggest Bg2 and Vg3 proteins are required for successful completion of embryonic development.

### Oocyte development

**Figure 3:**
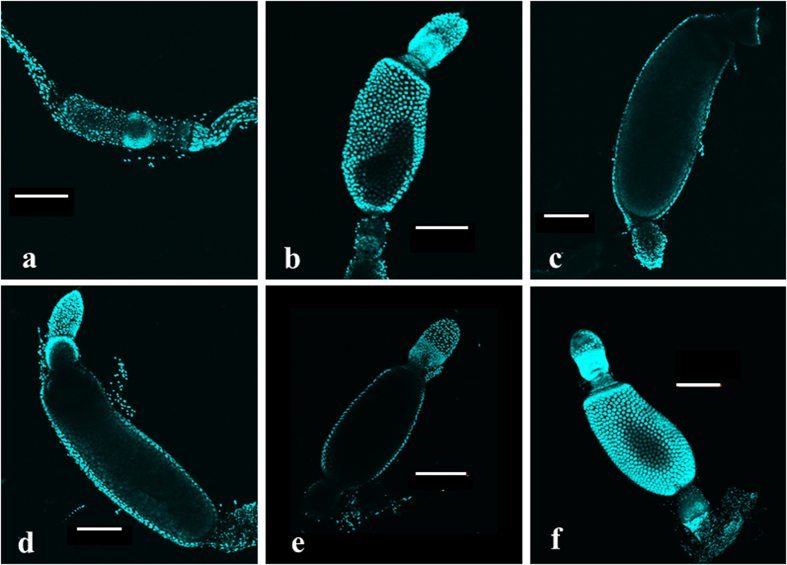
Effect of Vg knockdown on primary oocyte development. To study the effect of feeding and mating on the oocyte development, ovaries were dissected from adults on day 0, day 1, day 2, and day 3 after feeding and mating. The ovaries were stained with DAPI, and the primary oocytes were photographed under a confocal microscope. Oocytes grow progressively on day 0 (**a**), day 1 (**b**), day 2 (**c**) and day 3 (**d**) 3 after feeding and mating. Oocyte growth in unmated insects at three days after blood feeding is shown in (**e**). In insects injected with dsVg (dsVg1/2/3), the oocyte growth observed (**f**) is similar to that in insects that are fed but not mated.

To study the effect of feeding and mating on oocyte growth, ovaries were dissected from the female bed bugs at 24 h interval after feeding and mating. Only the most mature primary oocyte from each was observed. A gradual increase in the size of primary oocyte was observed from day 0 to day 3 (Fig. 3a–d). The maximum size of primary oocyte was observed on day 3 after feeding and mating. Primary oocyte length in the group of female adults which were not fed and not mated or only mated did not show any development and are similar to the oocytes in the newly emerged adults that were blood fed and mated (Fig. 3a). Only feeding also increased growth in the primary oocyte, however to a smaller size compared to fed and mated insects (Fig. 3e). Development of primary oocyte was reduced in Vg knockdown insects (Fig. 3f).

### Regulation of Vg gene expression

**Figure 4:**
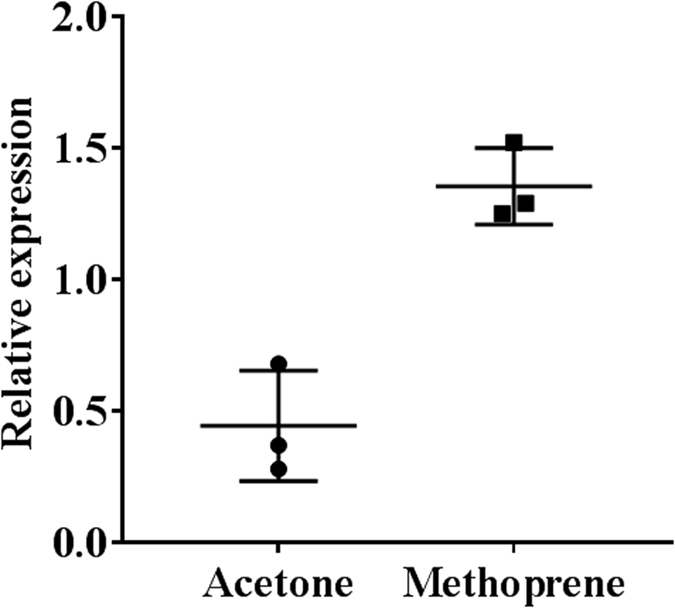
Effect of methoprene application on Vg gene expression. Ten μg methoprene in acetone or acetone alone was topically applied on day 0, day 1, day 2 and day 3 to fed adult virgin females. Total RNA was isolated on day 4 and Vg mRNA levels were determined after normalization with *ribosomal protein 8 (rpl8)*.

**Figure 5:**
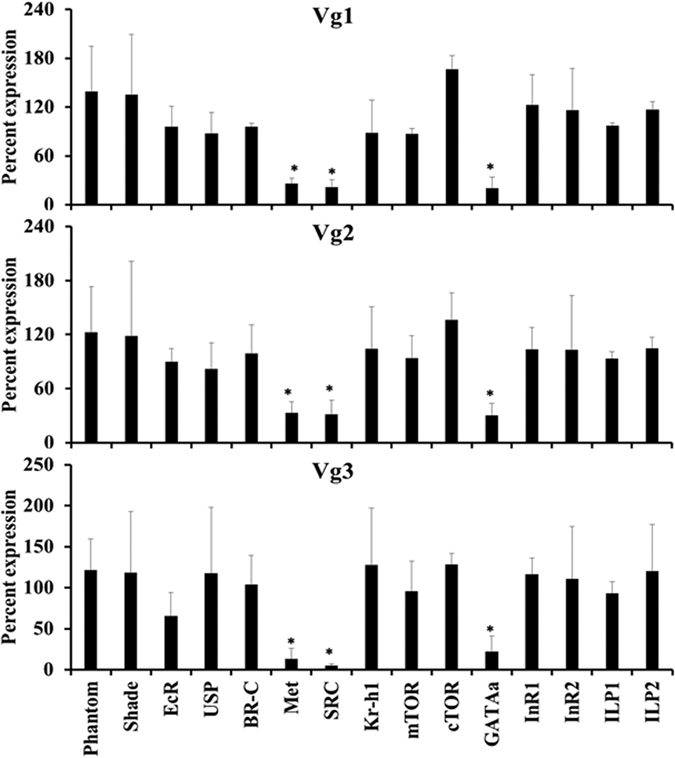
Regulation of Vg gene expression. To identify key genes involved in regulation of Vg gene expression, dsRNA targeting key genes in ecdysone (Phantom, Shade, EcR, USP, BR-C), JH (Met, SRC, Kr-h1) and nutrition (mTOR, cTOR, GATAa, InR1, InR2, ILP1 and ILP2) signaling pathways were injected into the virgin adult females. The insects were incubated for five days after which they were blood fed and mated. Two days after feeding and mating, the total RNA was isolated and used to determine relative levels of Vg1, Vg2 and Vg3 mRNA levels using RPL8 gene expression for normalization. Each bar represents the average and standard deviation of three biological replicates. Student t-test was performed (p ≤ 0.05). All experiment were repeated twice.

Methoprene was applied on blood fed virgin female bugs. Ten μg of methoprene was applied at every 24 h interval, and the samples were collected at 48 h and 72 h. Vg1 showed a three-fold increase in the fat body (Fig. 4). To study the regulation of Vg gene expression further, relative expression of Vg mRNAs was determined in the insects injected with dsRNA targeting genes coding for proteins involved in JH, ecdysteroid and nutrition pathways. Knockdown of JH receptor Met reduced expression of Vg1 to 26%, Vg2 to 33% and Vg3 to 13% (Fig. 5). Similarly, knockdown of SRC, the co-activator of Met reduced mRNA levels of Vg1 to 22%, Vg2 to 32% and Vg3 to 5.2%. Knockdown of transcription factor GATAa also reduced mRNA levels of Vg1 to 21%, Vg2 to 31% and Vg3 to 22%. Knockdown of Kr-h1, a transcription factor involved in the JH pathway did not decrease the expression of *Vg*. Knockdown of genes coding for enzymes involved in ecdysteroid biosynthesis or action including Phantom, Shade, EcR, USP and BR-C or genes involved in nutritional signaling, InR1, InR2, ILP1, ILP2, mTOR, and cTOR did not have any effect on *Vg* expression (Fig. 5).

### Effect of knockdown of the key genes involved in ecdysone, JH, and nutrition signaling on fecundity and hatching

**Figure 6:**
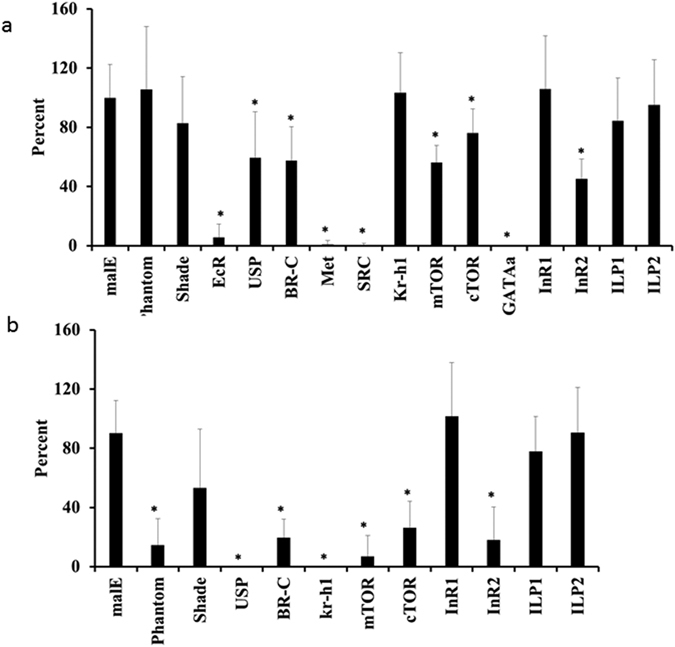
Effect of knockdown of the key genes involved in ecdysone, JH, and nutrition signaling on fecundity and hatching. Knockdown of key genes in ecdysone, JH and nutrition signaling was performed by injecting dsRNA targeting these genes into adult virgin females. These females were mated with untreated males. Percent fecundity and hatch rate, when compared to fecundity and hatch rate in control females injected with malE dsRNA, was calculated and plotted. Phantom and Shade, enzymes involved in ecdysteroid biosynthesis, ecdysone receptor (EcR), ultraspiracle (USP), broad complex (BR-C), methoprene-tolerant protein (Met), steroid receptor co-activator (SRC), Krüppel homologue 1 (Kr-h1), mammalian target of Rapamycin (mTOR), companion of TOR (cTOR), GATAa, insulin receptor 1 (InR1), insulin receptor 2 (InR2), insulin peptide 1 (ILP1), insulin peptide 2 (ILP2) were tested. Each bar represents percent values of average and standard deviation compared to the control. Percent fecundity of control or (malE) insects were set at 100%. The experiment was repeated twice with n ≥ 10. Student t-test was performed (p ≤ 0.05).

Knockdown of JH pathway genes, Met and SRC reduced fecundity to 0.7% and 0.5%, respectively (Fig. 6a). Knockdown of Kr-h1 had no effect on fecundity. Knockdown of ecdysone pathway genes, EcR, USP, and BR-C, reduced fecundity to 5.7, 59 and 57%, respectively. However, knockdown of Phantom and Shade had no effect on egg laying. Knockdown of mTOR, cTOR, InR2 and GATAa reduced egg laying by 56, 76, 45 and 0% respectively. However, InR1, ILP1, and ILP2 had no effect on fecundity (Fig. 6a).

Knockdown of ecdysone pathway genes, phantom, shade, USP, and BR-C reduced the hatching of eggs to 15, 53, 0 and 20%, respectively (Fig. 6b). The eggs laid by Kr-h1 knockdown insects showed 0% hatching. Knockdown of mTOR, cTOR and InR2 reduced hatching to 7, 26, and 18%, respectively. InR1, ILP1, and ILP2 knockdown did not show any effect on egg hatching (Fig. 6b).

### Expression analysis of genes in the fat body and ovary

**Figure 7:**
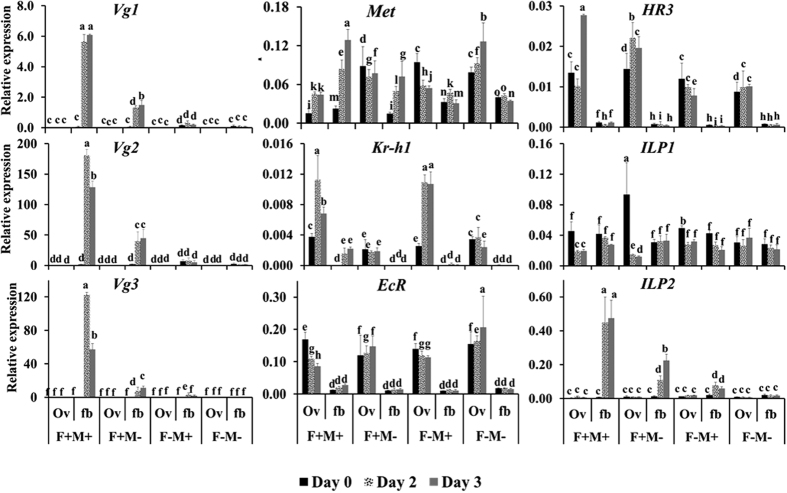
Expression of key genes involved in ecdysone, JH and nutrition signaling in the ovary and fat body. To study the effect of feeding and mating on the expression of these genes, four different treatments F+M+, adults fed and mated; F+M−, adults fed but not mated; F−M+, adults not fed, but mated; and F−M−, adults neither fed nor mated were included. The insects were dissected at day 0, day 2 and day 3 after feeding, total RNA was isolated from the fat body and ovaries. X-axis shows treatment groups and time, day 0 (black), day 2 (pattern), day 3 (gray) and the y-axis shows the relative mRNA levels. Relative mRNA expression of vitellogenin1 (Vg1), vitellogenin2 (Vg2), vitellogenin3 (Vg3), methoprene-tolerant protein (Met), krüppel homologue 1 (Kr-h1), ecdysone receptor (EcR), hormone receptor 3 (HR3), insulin peptide 1 (ILP1), insulin peptide 2 (ILP2), were determined after normalization with ribosomal protein 8 (rpl8). Data shown are mean + SD (n = 3). (Alphabet represents significance at 95% CI).

Three Vg mRNAs Vg1, Vg2 and Vg3 have been identified in *C. lectularius*. The expression patterns of all the Vg mRNAs were found to be similar and Vg mRNA was detected only in the fat body (Fig. 7). The highest levels of Vg mRNAs were detected in the fat body dissected from fed and matted females on the second and third day after feeding (Fig. 7) Feeding and mating of the female adults induced the expression of Vg gene. Vg mRNA levels reached a peak on day 2. Only feeding also induced expression of Vg gene but the mRNA levels are lower as compared to that in fed and mated insects. No increase in expression of Vg mRNA was detected in the unfed mated or unmated insects.

The mRNA of JH receptor Met was detected in both fat body and ovary. Met mRNA levels increased from day 0 to the maxim levels on day 3 in the fat body as well as in the ovary of the female bed bugs that were fed and mated (Fig. 7). Feeding appear to be an important trigger for an increase in Met mRNA levels in the fat body since mating did not cause an increase in the expression of Met mRNA over time (Fig. 7). Kr-h1 is expressed mainly in the ovaries. Kr-h1 expression increased on day two after feeding and mating. In ovaries, Kr-h1 expression peaked on day two. Only feeding and no mating as well as no mating groups did not show an increase in Kr-h1 expression in fat body or ovaries. Only mating group induced Kr-h1 gene expression in the ovaries but not in the fat body (Fig. 7).

Ecdysone receptor EcR mRNA showed an average of 6-fold higher expression in the ovaries in all treatment groups as compared to its levels in the fat body (Fig. 7). Ovaries constitutively expressed EcR. HR3, an early gene in the ecdysone pathway was expressed in all the groups tested at about 7-fold higher in the ovaries as compared to the fat body (Fig. 7). HR3 expression increased in the ovaries on day 3 after feeding and mating. HR3 expression remained unchanged in other treatment groups.

Expression analysis of genes involved in nutritional signaling showed that ILP1 is constitutively expressed in both the tissues in all the treatment groups (Fig. 7). However, ILP2 showed expression pattern similar to Vg. ILP2 expression peaked on day 2 and day 3 in the fat body dissected from fed and mated bugs. Only feeding was able to induce ILP2 in day 2 fat body. No expression of ILP2 was observed in the ovary (Fig. 7).

### Regulation of Oocyte development

**Figure 8:**
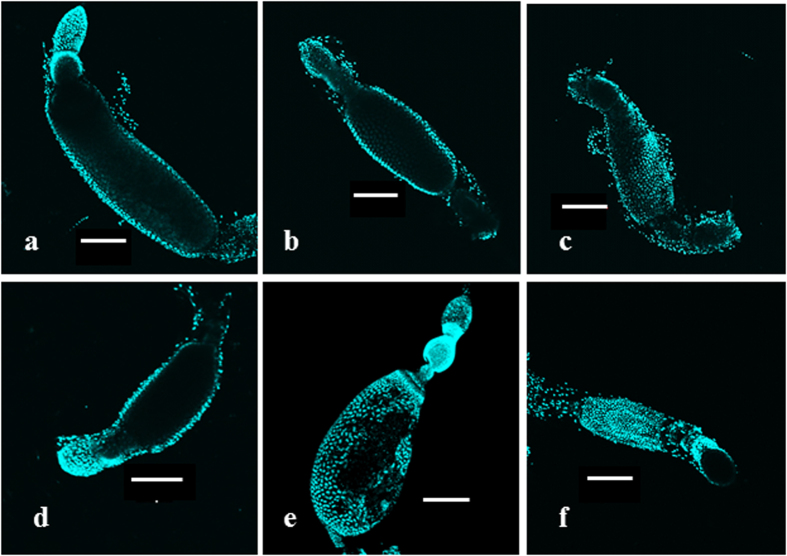
Oocyte development in adult females injected with dsRNA targeting key genes involved in ecdysone, JH, and nutrition signaling. To identify key regulators of oocyte maturation, adult females were injected with dsRNA targeting GFP (**a**), Met (**b**), SRC (**c**), EcR (**d**), BR-C (**e**) and GATAa (**f**). The ovaries were dissected on day 3 after feeding and mating of knockdown insects. The ovaries were stained with DAPI, and the primary oocytes were photographed under a confocal microscope.

Knockdown of JH pathway genes, Met and SRC, reduced the development of primary oocyte as compared to the oocytes in control insects (Fig. 8a–c). Similarly, knockdown of EcR and BR-C also decreased the length of the primary oocyte (Fig. 8d,e). Knockdown of nutritional signaling genes, transcription factor GATAa reduced oocyte length (Fig. 8f). These data suggest that JH, 20E as well as nutritional signals regulate oocyte development.

## Discussion

The results presented here identified three Vg genes and elucidated the hormonal regulation of expression of these genes. In *C. lectularius*, feeding and mating are prerequisites for initiation of reproduction in females. Both Vg mRNA and protein are not detected in nymphs as well as in the adults prior to feeding. Both Vg mRNA and protein levels increase after a blood meal (Figs 1 and 7). The mRNA levels of all three Vg genes increase beginning at two days after feeding and mating. However, in the animals that are fed but not mated, the Vg mRNA levels are significantly lower than that in the fed and mated animals. Very low levels of Vg mRNA were detected in unfed mated animals. Oocyte maturation requires Vg synthesis as well as mating as the unmated or Vg dsRNA injected animals showed the oocyte growth that is significantly lower than in the animals that are fed and mated (Fig. 3). Knockdown genes coding JH receptor Met and co-activator, SRC and transcription factor, GATAa reduced expression of all three Vg genes (Fig. 4), oocyte growth (Fig. 6) and a number of eggs laid by the females (Fig. 5). Also, knockdown the expression of EcR gene caused a reduction in oocyte growth as well as the number eggs laid by females. Taken together, these data suggest that nutritional as well as hormonal signals regulate female reproduction in *C. lectularius*. As reported in other insects including mosquitoes and cockroaches, feeding results in an increase in amino acid concentration and elevated levels of amino acids induce Vg synthesis. Nutritional signals work in concert with hormones to regulate vitellogenesis and oogenesis. *C. lectularius*, there is a likely increase in JH levels after feeding and mating contributed by either an increase in JH biosynthesis as previously reported in other insects including *Nilaparvata lugens*^24^ or by seminal fluids transferred from the males as reported previously in several insects including moth, *Heliothis virescens*^31^ and mosquito, *Aedes aegypti*^32^. Interestingly, in *H. virescens*, JH III transferred from the male induces biosynthesis of JH III in the females. The mechanisms that contribute to an increase in JH levels after feeding and mating in *C. lectularius* warrants further investigation. The major role of JH in the regulation of female reproduction observed in *C. lectularius* is in agreement with the previous reports on the major role for JH in the regulation of reproduction in *T. castaneum*^14^, Locusta migratoria^17^,^18^,^19^, *Blattella germanica*^20^,^21^, *Diploptera punctata*^22^, *Nilaparvata lugens*^24^, *Pyrrhocoris apterus*^23^ and other insects^16^.

Another interesting finding of these studies is the differences in the function of transcription factor Kr-h1 in transduction of JH signals. As reported in other insects including *Blattella germanica*^33^, *D. melanogaster*^34^*, Bombyx mori*^35^ and *Tribolium castaneum*^36^ in *C. lectularius* also JH suppression of metamorphosis is mediated by the transcription factor, Kr-h1^37^. However, JH regulation of female reproduction in this insect is not mediated by Kr-h1, as a knockdown in the expression of this gene did not affect Vg synthesis or fecundity. Interestingly, the conserved role of Kr-h1 in the regulation of embryogenesis^38^ has been observed in *C. lectularius.* Knockdown of Kr-h1 in adult females blocked embryonic development in eggs laid by these females. Similar differences in the Kr-h1 role in JH regulation of development and reproduction has been reported in in the linden bug, *Pyrrhocoris apterus*^23^. However, in *Locusta migratoria* Kr-h1 knockdown affects JH regulation of Vg gene expression and oocyte maturation^39^. In *C. lectularius* there may be other transcription factors that mediate JH regulation of Vg synthesis. Work is in progress to identify these transcription factors.

## Materials and Methods

### Insects

The bed bugs used in the present study are derived from the colony designated as NY1 raised from those collected in the infested apartment in Plainview, New York in April 2007. Insects were maintained at 26.7 °C, 65 ± 5% RH and a photoperiod of 14:10 h (L:D) and were fed defibrinated rabbit blood, supplied by the Quad Five Company, Montana, the USA based on the method proposed by Montes *et al.*^40^. Insects were fed once in a week. Fifth instar nymphs were collected after feeding. These nymphs develop into virgin adults in about 5–6 days, which were then used in the experiments described here.

### RNA Isolation, cDNA Synthesis, and Quantitative Real-Time PCR

Total RNA was isolated from three insects for each replicate using the TRI Reagent (Molecular Research Center Inc., Cincinnati, OH). The RNA was treated with DNase I (Ambion Inc., Austin, TX). cDNA synthesis (Promega) and qRT-PCR was performed using Applied Biosystems Step One Plus TM (Life Technologies TM Real-Time PCR System, Carlsbad, CA). FastStart SYBR Green Master (Roche Diagnostics, Indianapolis, IN) was used in a qRT-PCR reaction, with 0.2 μl of primers of 10 μM concentration in a 10 μl qRT-PCR reaction. Primer sequences are shown in the Table S1. The mRNA levels were normalized using the internal control, ribosomal protein L8 (RPL8) and the data were analyzed as reported previously^37^.

### Quantitative Real-Time PCR analysis of gene expression in whole body and tissue

Relative expression of selected genes was performed in whole body, ovaries, and fat body tissue samples. Fat body tissue also contained cuticle from abdomen attached to it. Whole insects were collected at day 0 to day 4 at 24 h interval after treatment. Insects were dissected at day 0, day 2 and day 3. The female bed bugs were provided with four treatments to study the effect of feeding and mating. The four groups were as follows:-blood fed and mated (F+M+), only blood fed (F+M−), only mated (F−M+), and without blood fed and not mated (F−M−). Total RNA was extracted, and qRT-PCR was performed.

### Double-stranded RNA synthesis, injection and mating experiments

Fragments of genes coding for target genes were PCR amplified using the gene-specific primers containing T7 promoter sequence on their 5′ ends (Table S1), and these DNA fragments were used to prepare dsRNA, as described by MEGA script RNAi Kit (Ambion Inc., Austin, TX). Day 1–2 old virgin adult females were used for the experiment. The insects were anesthetized with ethyl ether vapor for 2 min and lined on a glass slide covered with double-sided tape. One microgram of (0.2 μl of 5 μg/μl concentration) dsRNA was injected into the ectospermalege using nanojet. The glass needles were prepared by using needle puller (Idaho Technology, Salt Lake City, Utah). The dsRNA targeting GFP gene (prepared from a fragment of GFP gene amplified using EGFP vector purchased from Clontech laboratories as a template) was injected as a control. Injected adults were removed from the slide after recovery and kept in an incubator for five days before feeding them with rabbit blood. The uninjected virgin adult males were also blood fed. Engorged male and female insects were kept in a 48-well plate for two days. The males were then discarded.

### Methoprene application

10 μg methoprene in 1 μl acetone was applied on the abdomen of each adult virgin female after blood feeding. Methoprene application was repeated at 24 h and 48 h after first treatment. The control insects were treated with the same volume of acetone. The bugs were then kept in an incubator, and the samples were collected at 6 h after the final application.

### Dissection and confocal microscopy

The adult females were dissected in 0.01 M phosphate buffer saline (PBS) on the third day after feeding and mating to study the development of their primary oocytes. The ovaries were then fixed in 4% paraformaldehyde (in 1× PBS at pH 7.2) overnight. The tissue was rinsed with PBS and stained with DAPI. Pictures were then taken with a confocal microscope under illumination with light at 405 nm wavelength.

### Protein extraction, SDS-PAGE, and sequencing

Total protein from the hemolymph was extracted three days after treatment. Holes were made in the abdomen of the insect using a needle. The insect was kept in 20 μl PBS containing proteinase inhibitor and centrifuged at 1000 rcf for 1 minute. Total protein content in the supernatant was quantified using Bradford assay^41^. After denaturing the protein samples, 10 μg of the sample was loaded on a 6% SDS-PAGE. The gel was stained using Coomassie brilliant blue dye. The gel image was recorded using a gel documentation system. The gel was submitted to Proteomics Core Facility, the University of Kentucky for the identification of ~220 KDa band. The samples were digested with trypsin. The extracted peptides were desalted and analyzed by MALDI TOF/TOF.

### Statistical analysis

Statistical analysis was performed using Statistx 10.0. Student t-test (unpaired t-test) was performed for comparing significance in knockdown and induction of gene expression. One way ANOVA followed by Tukey HSD test was performed for determining the significance of expression profiles of genes.

## Additional Information

**How to cite this article**: Gujar, H. and Palli, S. R. Juvenile hormone regulation of female reproduction in the common bed bug, *Cimex lectularius. Sci. Rep.* **6**, 35546; doi: 10.1038/srep35546 (2016).

## Supplementary Material

Supplementary Information
