# Supplementary material for: Juvenile hormone regulation of female reproduction in the common bed bug, Cimex lectularius
Source: Sci Rep. 2016 Oct 20;6:35546. doi: 10.1038/srep35546 (PMC5071884; doi:10.1038/srep35546)
Supplement: Supplementary Information [file srep35546-s1.pdf]

Juvenile hormone regulation of female reproduction in the common bed bug, *Cimex lectularius*

Hemant Gujar and Subba Reddy Palli

Figure 1S

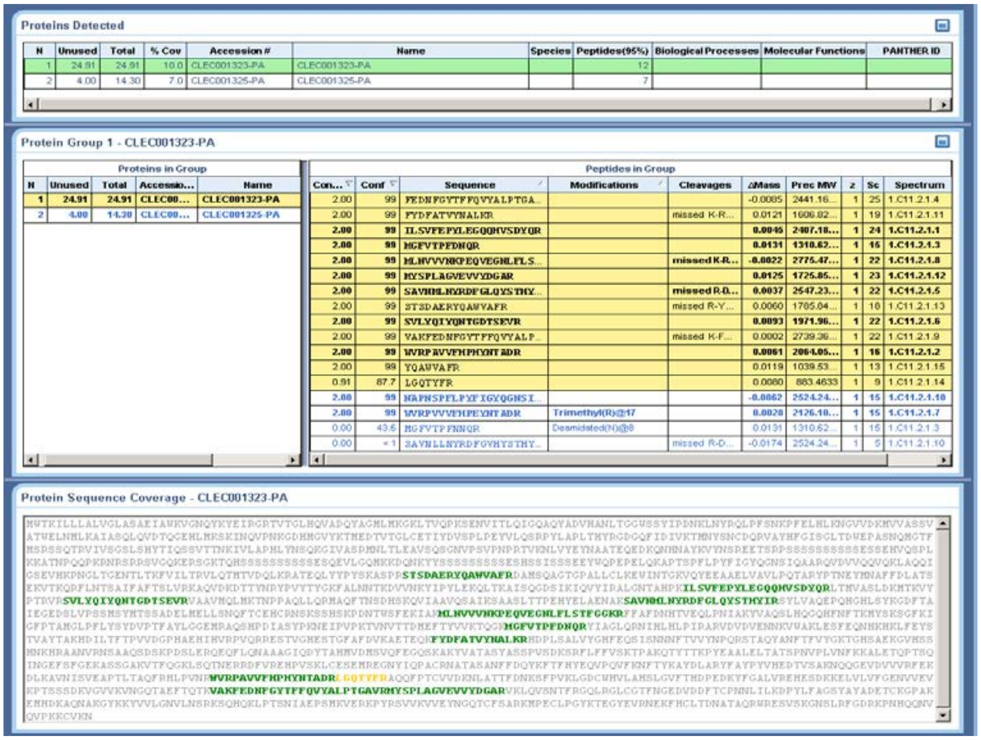
Proteins Detected
Protein Group 1 - CLEC001323-PA
Proteins in Group
Peptides in Group
Protein Sequence Coverage - CLEC001323-PA

(a)

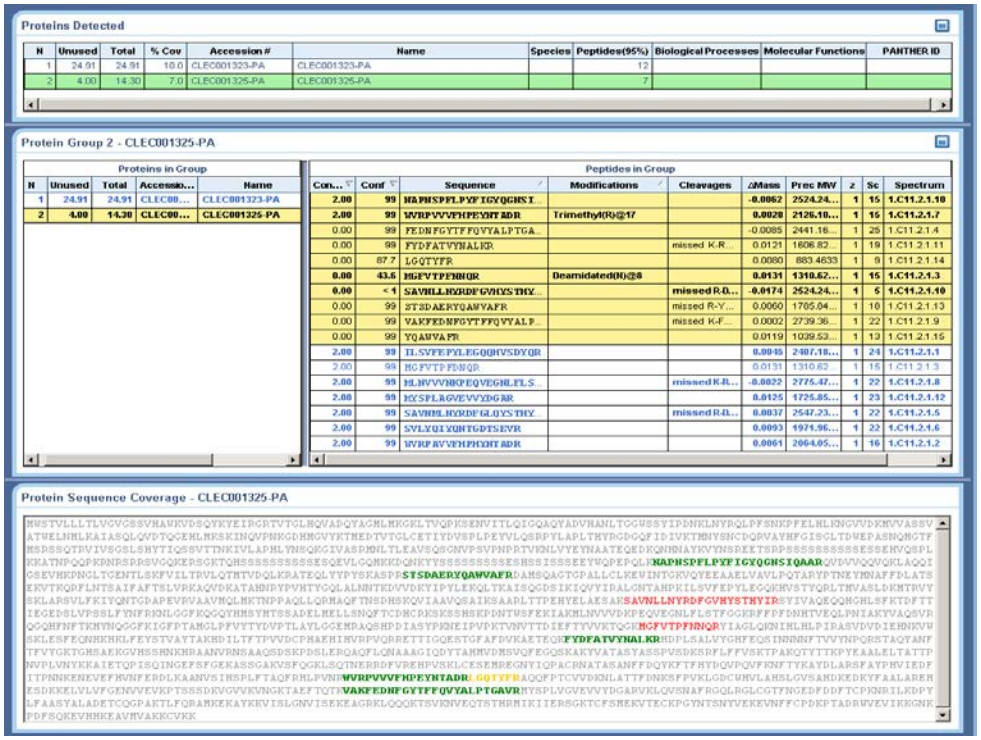
Proteins Detected
Protein Group 2 - CLEC001325-PA
Proteins in Group
Peptides in Group
Protein Sequence Coverage - CLEC001325-PA

(b)

Fig. S1: Identification of vitellogenin protein by MALDI TOF/TOF. Peptides sequenced matched with CLEC001323 (Vg2) and CLEC001325 (Vg3) proteins.

Supplementary Table 1: List of primers used in this study

dsRNA primers	Forward primer	Reverse primer
Vg1	CTACGGACAAGACGAAGAATTT	CTTACTGGTGTACAGGTTGTTT
Vg2	GTTCTCGGAAACGTCCTTAACA	CGCATTTCTTGGGTACTTGAAC
Vg3	CCATGAAAGAGAAAGCTTACAA	CTGGGAGAAGTCTGGTTTATTT
Phantom	ACATCACACTGACTGAACTGGCG	GAAACGTCGCTGCTCCTTCCATTT
Shade	CCTTGGAAAGCTCAACTCGTCCT	TGTGCTTTGTCATACTTGGCTGGC
EcR	TATGTAGACAACAGGGCAAGGCC	GGAAACGTCTTCTGGCACGGATT
USP	TGAGGAGTTCATTCCAACCCGCAT	TCTATGGGCATGAAACGTGAGGCT
BR-C	TGAGGCAAAGGTCTCTTTCGTCGT	TTCATCGGCATTGCATTGTCCTGG
Met	CCAAGCTCGACCAGAGAGATAAGA	AGCTGCTCCGTGCTTGTAT
SRC	AGCAAAGAGGAGGTCCTAGGGAAA	CATATTTGCACACTCGCTGCTGCT
Kr-h1	TTCGGATAACAACCTCCGCTTGGA	GGTCCACAATCGGAGTCACACA
mTOR	ATGGCAGGAGAAAGTTACCAGCGA	AAGAGGCGTACTTAAGCCAGGTGT
InR1	GTGTTCCCGAACTTGGCCGTTATT	AAACACAATTGGACGGACCACTGC
InR2	GCTCCTTATATACCATCTGAACC	CTTGGAATCCTTCTCCCATAAC
ILP1	TTCTGACCGCTCACAACCGATGA	TCCCTCGCTGCAGTACGATTCAA
ILP2	CCTATAATTTCGAGGATGATCGTATGAATG	CATCCTTGACCATCAGCACAATAGG
cTOR	CAACTGAAGAAGACACTCCTAATA	GGATCTACTCTTTCTACCACTTTC
GATAa	AGCAGTCGGTTACAGCATCAGGAA	TGTACATCGCGGTTGTGTAGTCGT
qRT-PCR	Forward primer	Reverse primer
Vg1	CGTGTTGAGCTTTACGCGAGCAAT	ACCTGTCGGTCGTCATGAAATCGT
Vg2	TATGTTCATGAAGACACCGTTT	GCCTCGACTGAGATGTTGACG
Vg3	CTCATGTTATTGAAGATTTTATTACCCCT	GAGAATGGATGGAGACATTAGCA
Phantom	TAATCGGCATTGTAGGGCCTGTCA	CTTTCAGGTAGCGCAACAAACGGT
Shade	TCCAAATCGCTTCTTCCCTCACGA	GCGAACAACAAAGCCCTTGACGAT
EcR	ATCCTCTTCGCCAATAACCAGCCT	TGCCTGCAGAACCTAAGTAGACCT
USP	TGTGTCGACAGAAACGTCACCGAT	TGTTAAGGTTGCCCTCGCTTAGGT
BR-C	ATCGCCAAGGGAAGACATGGAAGA	TGGAGTCTTCCCGCTCATTGTGAA

Met	TCGTCCCTGGTCACAGAAACGAAA	ACTGCGGTTGATCGCTCTTCGATA
SRC	ATGAGCTCACTTTCCGTCAAGCCT	GGTCGCAGATGCTTGCTGTTCTTT
Kr-h1	ACGCTTTGGCGTACTGAATAACGG	TTTCGGGATCGCCGATTTCTGTCT
mTOR	AAGGATGGCTCCCGATTATGAGCA	CCAAATCATCGCCTTGCGTGTGTT
InR1	AGACGGCAGATGAGCTTGCTAAGT	AATGGAGGCTGTTCAAGGGTCTCA
InR2	GACTGAACTGATAGCTGAAGTAA	CCTCAATTGAATAGACAACATATCC
ILP1	AACGTGGCTACAACATGGCGTTTC	ATCCCTCGCTGCAGTACGATTCAA
ILP2	GTGGCCGGACTTTAACGAAGATGT	ATCCATTGCAAATGACTCGGAGCG
TOR	GGACAATGGCCTCAAGTGTATGAAGC	ACCAATCGACCGACAAAGGTACGA
cTOR	CCACCTATCACCTATCAACATATC	ATCTAGAAGCTGGGTGTAAATTC
GATAa	ACTAGAGAGCAAAGTCTGGCAGCA	CCAATGCGTCGGCCATAGTGAAA
HR3	TTGACCCTGTCAACGAGACAAGC	ATCACATGCGAGGGTTGCAAGG
